# The AcrAB efflux pump contributes to the virulence of Enteroaggregative *E. coli* by influencing the aggregative behavior

**DOI:** 10.3389/fcimb.2025.1633585

**Published:** 2025-08-07

**Authors:** Martina Laudazzi, Emily Schifano, Francesca Sivori, Ludovica Altieri, Daniela Uccelletti, Enea G. Di Domenico, Bianca Colonna, Martina Pasqua, Gianni Prosseda

**Affiliations:** ^1^ Department of Biology and Biotechnologies “Charles Darwin”, Institut Pasteur in Italy, Sapienza University of Rome, Rome, Italy; ^2^ Microbiology and Virology, San Gallicano Dermatological Institute, IRCCS, Rome, Italy; ^3^ Institute of Molecular Biology and Pathology (IBPM), CNR National Research Council, Rome, Italy

**Keywords:** multidrug efflux pumps, AcrAB, enteroaggregative *Escherichia coli* (EAEC), *E. coli* pathogens, bacteria-host interactions, bacterial transmembrane complexes

## Abstract

Multidrug efflux pumps play a major role in the emergence of antibiotic resistance. AcrAB is particularly important among them, as it is the main RND pump in *Escherichia coli* and other *Enterobacteriaceae*. In addition to contributing to multidrug resistance, AcrAB also plays a significant role in the virulence of several pathogens. Here, we report that AcrAB contributes to both adhesion to host cells and biofilm formation in EAEC, an enteropathogenic group of *E. coli* known to cause both acute and persistent diarrhea. EAEC is an emerging pathotype of *E. coli* characterized by its ability to adhere extensively to epithelial cells in an aggregative manner and to form voluminous biofilms, which favor infection persistence. We found that the deletion of *acrB* prevents biofilm formation and reduces the export of extracellular DNA (eDNA). By using a specific inhibitor of AcrB, we confirmed the requirement of AcrB transporter activity for biofilm biogenesis. The characteristic aggregative pattern of EAEC is also strongly impaired in the absence of AcrB or in the presence of an efflux-defective AcrB D408A transporter, while it is restored in the Δ*acrB* strain complemented with *acrB*. Finally, we show that the EAEC 17-2 Δ*acrB* derivative is significantly less lethal than the wild type in *Caenorhabditis elegans*. Complementation with the *acrB* gene, but not with the *acrB*_D408A_ allele, fully restores the virulence phenotype after infection. Overall, our results confirm the relevance of the AcrAB efflux pump as a virulence determinant and contribute to understanding the mechanisms adopted by EAEC to form thick biofilms and copious adherence to the epithelial cells, both features enhancing persistence during infections.

## 1 Introduction

Antibiotic-resistant infections pose a serious concern to global public health. In particular, ESKAPE organisms, together with pathogenic *Escherichia coli* (collectively referred to as ESKAPEE), are responsible for the large majority of global deaths attributable to resistant pathogens (World Health Organization, 2017). A critical role in the emergence of drug resistance is played by multidrug resistance (MDR) efflux pumps (EPs) - membrane protein complexes capable of exporting all clinically relevant classes of antibiotics used to treat infections (Nikaido and Pagès, 2012; Blair et al., 2015; Li et al., 2015). Several studies have highlighted the ability of the MDR efflux pumps to also transport a variety of structurally diverse compounds, such as endogenous bacterial metabolites, quorum-sensing molecules, and fatty acids, conferring them other important roles in the physiology of the bacterial cell. These include stress response, interactions with plant and animal hosts, maintenance of cellular homeostasis, interbacterial communication, and biofilm formation (Alcalde-Rico et al., 2016; Alav et al., 2018; Henderson et al., 2021; Pasqua et al., 2021). Based on their structure, energy source, and type of exported substrate, MDR EPs are classified into seven major families (Alav et al., 2021). Among these, the resistance-nodulation-division (RND) family is of particular interest for antimicrobial resistance due to its unusually large spectrum of substrates (Du et al., 2018; Zwama and Nishino, 2021). RND efflux pumps share a conserved tripartite architecture, comprising an active proton antiporter in the inner membrane, a channel protein spanning the outer membrane and periplasm, and a periplasmic adaptor protein that connects the transporter to the channel protein. AcrAB-TolC is regarded as the most relevant RND MDR EPs in many bacteria, including *Enterobacteriaceae*, due to its high abundance and capability to export a wide variety of compounds, including bile salts (Piddock, 2006; Du et al., 2018; Alav et al., 2021). The crystal structures of all three components of AcrAB-TolC EP have been resolved, supporting the model that these proteins assemble into a channel that expels substrates from the cytoplasm into the external environment (Murakami et al., 2002; Du et al., 2014). The AcrB transporter is a homotrimeric protein associated with six periplasmic AcrA proteins. During active substrate transport, AcrB cycles through three distinct conformations: Loose, Tight, and Open. Each AcrB monomer features two distinct drug-binding pockets i.e. a deep distal binding pocket (DBP) and a proximal binding pocket (PBP), separated by a switch loop (Du et al., 2014; Zwama and Nishino, 2021). The AcrA adaptor extends into the periplasm while remaining anchored to the cytoplasmic membrane via an N-terminal lipid domain. It plays a crucial role in maintaining the stability of the entire EP and transmits conformational changes from AcrB to the TolC outer membrane channel, facilitating its opening (Alav et al., 2021; Zwama and Nishino, 2021).

AcrAB also contributes to the pathogenesis and virulence of several human and plant pathogens (Piddock, 2006; Blanco et al., 2016; Du et al., 2018; Henderson et al., 2021). In *Salmonella typhimurium*, the absence of a functional AcrB transporter impairs the invasion of intestinal epithelial cells and murine macrophages and attenuates virulence in a mouse infection model (Buckley et al., 2006; Webber et al., 2009; Wang-Kan et al., 2017). Similarly, the loss of AcrAB in *Klebsiella pneumoniae* reduces its ability to cause pneumonia in mice (Padilla et al., 2010), while in *Enterobacter cloacae*, it compromises the capacity to induce systemic infections (Pérez et al., 2012). It has been reported that in *Moraxella catarrhalis*, a functional AcrAB-OprM is required for the invasion of human nasopharyngeal cells (Spaniol et al., 2015). More recently, it has been shown that in *Shigella flexneri*, the etiological agent of bacillary dysentery, the deletion of both *acrA* and *acrB* reduces survival within epithelial cells and prevents spreading to adjacent cells (Coluccia et al., 2023). Similarly, in Adherent-Invasive *Escherichia coli* (AIEC), a group of pathogenic *E. coli* highly abundant in the ileal mucosa of Crohn’s disease patients, the AcrAB pump strongly influences the pathogenicity process by contributing to bacterial viability inside macrophages (Fanelli et al., 2023).

Enteroaggregative *E. coli* (EAEC) is an emerging group of pathogenic *E. coli*, strongly involved in gastrointestinal infections and responsible for severe diarrheal disease in developing and developed countries (Nataro, 2005; Pokharel et al., 2023). EAEC strains are known for their ability to form voluminous biofilms, embedded in a thick mucus layer, on the surface of enterocytes (Estrada-Garcia et al., 2014). Indeed, EAECs adhere to each other as well as to the cell surface, forming an aggregative adherence pattern (AA) (known as “stacked bricks”), where bacteria are elongated and sometimes line up in a single layer on the cell surface. This characteristic phenotype is mediated by several genes located on a large virulence plasmid (pAA plasmid), which encodes factors involved in the biogenesis of aggregative adhesion fimbriae (AAFs), the key proteins responsible for cell adhesion during EAEC infection (Berry et al., 2014; Ellis et al., 2020). In addition to forming the characteristic mucoid biofilm adherent to the intestinal mucosa, EAEC causes diarrheal disease by inducing cytotoxic effects on the enterocyte brush border, through the release of toxins, which induce the host’s inflammatory response (Ellis et al., 2020; Petro et al., 2020).

As discussed above, AcrAB has been demonstrated to significantly contribute to the virulence of several pathogens belonging to the *Enterobacteriaceae* family, such as *Salmonella*, *Shigella* and AIECs, as well as to be involved in biofilm formation. Unlike previously studied *E. coli* pathotypes, which invade epithelial cells and adopt an intracellular lifestyle, EAEC is non-invasive but gives rise to a unique aggregative adherence pattern sustained by a robust biofilm. In light of these key traits of EAEC pathogenesis, this study aimed to investigate the potential contribution of the AcrAB efflux system to its virulence phenotype. To this end, we analyzed biofilm formation and the ability to adhere to epithelial cells of the EAEC strain 17–2 mutants deleted of *acrA* or *acrB*. Moreover, the infection capability of these mutants was evaluated in the *Caenorhabditis elegans in vivo* model. Our results clearly indicate that a functional AcrAB is required for the full expression of the EAEC pathogenicity in the colonization of the host cells.

## 2 Materials and methods

### 2.1 Construction of bacterial strains and plasmids

Bacterial strains and plasmids used in this study are described in **Supplementary Tables S1**, **S2**. Strain 17–2 is a clinical isolate of EAEC. Cloning was performed using *E. coli* DH10b as the host strain. The deletion mutants, 17-2 Δ*acrA* and 17-2 Δ*acrB*, were obtained using the one-step method of gene inactivation (Datsenko and Wanner, 2000). Specifically, the kanamycin resistance gene was amplified from the pKD4 or pKD13 plasmids using the oligo pairs AFW/ARV and BFW/BRV for *acrA* and *acrB* deletion, respectively. The resulting amplicons were electroporated into the 17–2 pKD46 strain, which expresses the Red recombinase. The obtained mutants were then deprived of Km resistance by transformation with the pCP20 plasmid, which encodes a flippase that facilitates flippase/flippase recognition target (Flp/FRT) recombination (Datsenko and Wanner, 2000). The pEA*acrB* plasmid was constructed by cloning the *acrB* gene under the control of the tac promoter from the pGIP7 plasmid, using the oligo pair pEA*acrB*FW/pEA*acrB*RV. Plasmid pEA*acrB* served as the template for site-directed mutagenesis. As previously described (Fanelli et al., 2023), GeneArt^™^ Site-Directed Mutagenesis System (GeneArt^™^ Invitrogen-Thermo Fisher Scientific, Waltham, MA, USA), was employed along with the oligo pair pEA*acrB*_D408A_FW/pEA*acrB*_D408A_RV to introduce a codon change from GAC to GCC (D408A), replacing the A at position 1223 (relative to the ATG of *acrB* gene) with a C.

### 2.2 General procedures and growth conditions

All bacterial strains were grown aerobically in Luria-Bertani (LB) (Sigma-Aldrich, St. Louis, MO, USA) medium at 37°C with shaking at 200 rpm, unless otherwise specified. Solid media contained 1.6% agar. Antibiotics (Sigma-Aldrich, St. Louis, MO, USA) and inhibitors were used at the following concentrations: kanamycin at 30 μg/mL, chloramphenicol at 25 μg/mL, ampicillin at 50 μg/mL and 1-(1-naphthyl-methyl)-piperazine (NMP) at 100 μg/mL. Growth kinetics of 17–2 and its derivatives in different media (LB and DMEM) were measured using a CLARIOstar plate reader (BMG LABTECH, Offenburg, Germany).

The mutations and constructs obtained in this study were confirmed using PCR reactions, routinely performed with DreamTaq DNA polymerase (Thermo Fisher Scientific, Waltham, MA, USA), and verified by DNA sequencing (Biofab, Rome, Italy). For amplifying coding sequences necessary for cloning, Pfu Taq DNA polymerase (Thermo Fisher Scientific, Waltham, MA, USA) was used. The sequences of the oligonucleotides, designed based on the 17–2 genome, are listed in **Supplementary Table S3**. DNA and protein sequence comparisons were conducted using the NCBI BLAST tool (http://blast.ncbi.nlm.nih.gov/Blast.cgi).

### 2.3 Antimicrobial susceptibility testing

Minimum inhibitory concentrations (MICs) were determined using a broth microdilution method in 96-well microplates (Euroclone S.P.A., Milan, Italy – 96 well flat bottom). EAEC 17–2 wt strain and its derivatives 17-2 Δ*acrA*, 17-2 Δ*acrB*, 17-2 Δ*acrB_*pEA*acrB* and 17-2 Δ*acrB_*pEA*acrB*_D408A_ were grown in LB until an OD_600_ of 0.5-0.7, corresponding to the exponential phase of growth. Each bacterial suspension was then diluted to OD_600_ 0.02 in LB and 100 µL aliquots were inoculated in each micro-well into 100 μL of 2-fold serial dilutions of each antibiotic (erythromycin 0.5 µg/mL to 256 µg/mL, tetracycline 0.0313 µg/mL to 16 µg/mL). 1-(1-naphthyl-methyl)-piperazine (NMP) (Sigma-Aldrich, St. Louis, MO, USA) was dissolved in DMSO and tested from 1.25 µg/mL to 1280 µg/mL in BHI (Becton Dickinson, USA) medium (**Supplementary Figure S3**). Bile salts (B8756, Sigma-Aldrich, St. Louis, MO, USA), consisting of an approximate 1:1 mixture of cholate and deoxycholate, were used at 0.16% (w/v). After an incubation of 18 h at 37°C, results were collected by measuring OD at 600 nm using a CLARIOstar plate reader (BMG LABTECH, Offenburg, Germany). The MIC value considered was the lowest drug concentration that exhibited complete inhibition of microbial growth. A 2-fold or greater decrease in MIC values was considered significant. The results are averages of at least three independent experiments.

### 2.4 Biofilm quantification and characterization

Biofilm formation was assessed in *E. coli* strains using crystal violet (CV) staining, CFU quantification, and extracellular DNA (eDNA) measurement, as previously described (Sivori et al., 2024). 100 µL of bacterial suspension (10^5^ CFU/mL) were cultured in 96-well polystyrene plates at 37°C under static conditions for 24 h. Biofilm biomass was quantified by staining with 0.1% CV or 0.01% CV when combined with NMP, ethanol-acetone (4:1) solubilization, and absorbance measurement at 570 nm (Multiskan SkyHigh, Thermo Fisher Scientific, USA). Viable cell counts were determined by plating serial dilutions on MacConkey agar (BioMérieux, France). After biofilm formation, wells were washed, resuspended in sterile water, and scraped for CFU enumeration. eDNA quantification was performed by adding Tris-EDTA buffer (Sigma-Aldrich, St. Louis, MO, USA) and freshly prepared PicoGreen solution (Thermo Fisher Scientific, Waltham, MA, USA), followed by fluorescence measurement (excitation: 485 nm; emission: 535 nm; Wallace Victor 3, PerkinElmer). A lambda DNA (Thermo Fisher Scientific, Waltham, MA, USA) standard curve was used for quantification. Experiments were conducted in triplicate and repeated three times (Sivori et al., 2022).

For biofilm metabolic activity, bacterial suspensions were allowed to adhere for 5 h before removal of non-adherent cells. Resazurin solution (Promega, USA) was then added, and plates were incubated for an additional 20 h. Absorbance was measured at 570 nm using the Multiskan SkyHigh reader, with absorbance readings taken every 20 min (Sivori et al., 2022).

To characterize the protein component of the biofilm matrix, biofilms formed in BHI under static conditions for 20 h were stained with 100 µL of FilmTracer SYPRO Ruby (Invitrogen, Thermo Fisher Scientific, Waltham, MA, USA) for 30 min at room temperature. After two washes with sterile distilled water, fluorescence intensity was measured (excitation: 485 nm; emission: 625 nm; Wallace Victor 3, PerkinElmer) (Ravaioli et al., 2020). Amyloid fiber content was quantified using EbbaBiolight (Ebba Biotech AB, Sweden). 200 µL of bacterial cultures were incubated under static conditions at 37°C in BHI supplemented with 1,000-fold diluted dye. After 24 h, fluorescence was measured (excitation: 535 nm; emission: 660 nm) using the Wallace Victor 3 plate reader (Choong et al., 2016).

### 2.5 Cell cultures and HEp-2 adherence assays

The HEp-2 cell line (CCL-23, American Type Culture Collection, Manassas, VA, USA) was grown in Dulbecco’s Modified Eagle Medium (DMEM) (Gibco, Thermo Fisher Scientific, Waltham, MA, USA) supplemented with 10% heat-inactivated fetal bovine serum (FBS) (Gibco, Thermo Fisher Scientific, Waltham, MA, USA), 0.05 I.U./mL penicillin and 0.05 I.U./mL streptomycin (Sigma-Aldrich, St. Louis, MO, USA). Cells were maintained at 37°C in a humidified atmosphere with 5% CO_2_.

The adherence assay protocol was adapted from that of Cravioto et al., 1979. Briefly, HEp-2 cells at a concentration of 2 x 10^5^ cells/mL were seeded into 30 x 10 mm sterile plastic tissue culture Petri dishes (Nunc) into which a sterile rectangular coverslip had been placed. Cells were then incubated overnight and grown to 60% confluency. Two hours before bacterial infection, the culture medium was replaced with fresh DMEM. At the time of infection, HEp-2 cell monolayers were washed three times with 1X PBS and then overlaid with exponential cultures of EAEC strains diluted in DMEM containing 1% D-mannose, which was added to inhibit adhesion due to type I pili. This resulted in a final multiplicity of infection of 100. The infected cells were incubated for 24 h, after which they were carefully washed twice with 1X PBS and fixed with 4% paraformaldehyde for 20 min. Following fixation, the samples were washed three times with 1X PBS and permeabilized in 0.5% Triton X-100 for 10 min, then washed twice again. The samples were stained with 4’,6-diamino-2-phenylindole (DAPI, 0.5 µg/mL) for 5 min and washed twice. Finally, samples were stained with Evans Blue (0.01%) for 30 sec and then carefully washed with 1X PBS. Coverslips were mounted using 10 µL of a 1:1 PBS-glycerol solution. Images were acquired at the IBPM imaging platform https://www.imagingplatformibpmcnr.it with a Nikon Ti2 confocal spinning disk microscope (implemented with Crest X-Light V3 module from CrestOptics) equipped with the Kinetix sCMOS camera (Teledyne Photometrics), a 60× (immersion oil, NA 1.4) objective and CELESTA lasers (Lumencor). Image acquisition and analyses were performed using NIS-Elements AR software modules (Nikon). 2D image projections from z-stacks were created using the Maximum Intensity Projection (MIP) function. 3D reconstructions were created using the 3D Volume View function.

For quantitative adherence assays, after infection and 24 h incubation, HEp-2 monolayers were washed three times with 1X PBS. Each well was treated with 1% Triton X-100 for 20 min and serial dilutions of lysed sample were plated out for viable counting on LB, with antibiotics added where necessary. At least three independent assays were performed.

### 2.6 *C. elegans* killing assay

For *C. elegans* infection, bacterial lawns were prepared by spreading 30 μL of each overnight bacterial culture, corresponding to 1 × 10^8^ cells, onto Nematode Growth Medium (NGM) agar plates (35 mm). The plates were incubated at 37°C for 24 h to allow bacterial growth before being seeded with young adult nematodes grown at 16°C from a synchronized culture (Schifano et al., 2019). Plates seeded with the standard food bacterium *E. coli* OP50 served as controls. Each day, 80 worms per condition were transferred to new plates, plated with fresh bacterial cultures, and monitored under a stereomicroscope (Olympus SZ30, Olympus Optical Co. Ltd., Tokyo, Japan). The infections were performed at 25°C and the number of dead worms was recorded daily. A worm was considered dead if it failed to respond to touch with a platinum wire; dead worms stuck to the wall of the plate were excluded from the analysis. Mortality data from duplicate plates were pooled and subjected to survival analysis. The results shown are representative of repeated independent assays.

For intestinal lumen diameter analysis in *C. elegans*, 10 synchronized worms per condition, after 72 h of infection, were washed in M9 buffer, mounted onto 3% agarose pads containing 20 mM sodium azide and observed with a Zeiss Axiovert 25 microscope. Intestinal lumen diameter was measured by using Zeiss ZEN Microscopy Software 2011.

### 2.7 Statistical analysis

The statistical differences were assessed using GraphPad Prism 9.0, which calculated p-values using a one-way ANOVA or, when appropriate, a two-tailed student’s *t*-test. For *C. elegans* tests, survival data were analyzed using the Kaplan-Meier method. Survival curves were generated using GraphPad Prism 9.0, and statistical differences between groups were assessed using the log-rank (Mantel-Cox) test. Data from duplicate plates were pooled for analysis, and results are representative of at least three independent experiments. Statistical significance was set at *p* < 0.05.

## 3 Results

### 3.1 Silencing of *acrA* and *acrB* affects resistance to antibiotics and bile salts in EAEC 17-2

Comparative in silico analysis revealed the presence of genetic loci encoding the 20 most relevant EPs associated with multidrug resistance in the EAEC 17–2 genome. The *E. coli* MG1655 genome was used as a reference (Nishino and Yamaguchi, 2001; Teelucksingh et al., 2022). Unlike other genera, such as *Shigella*, which underwent a strong gene decay in the evolutionary process from commensal to pathogen (Feng et al., 2011), we found that EAEC 17–2 retained the genes coding for all MDR EPs, including AcrAB. This analysis also highlighted the conservation of a high degree of sequence homology (**Table 1**).

**Table 1:** Analysis of MDR EP encoding genes present in the 17–2 genome.

Efflux pump family	ABC	MFS										RND						SMR		MATE
EPs in *E. coli* MG1655	*macAB*	*bcr*	*emrAB*	*emrKY*	*mdtG*	*mdtH*	*mdtL*	*emrD*	*fsr*	*mdfA*	*mdtM*	*acrAB*	*acrD*	*acrEF*	*cusB*	*mdtABC*	*mdtEF*	*emrE*	*mdtJI*	*mdtK*
EPs in EAEC 17-2	+ 99.97%	+ 100%						+ 99.92%			+ 95.54%	+ 100%	+ 99.97%	+ 99.98%	+ 98.12%	+ 99.38%	+ 99.91%	+ 91.89%	+ 100%	+ 100%

The genomes of *E. coli* MG1655 and EAEC 17–2 strain were compared using NCBI BLAST (http://blast.ncbi.nlm.nih.gov/Blast.cgi). To obtain the nucleotide sequences of each MDR EP encoding gene, EcoCyc was used as a reference database (https://ecocyc.org). (+) MDR EP encoding genes present in the 17–2 genome. Sequence homology is specified as a percentage. Efflux pumps families’ acronyms: ATP-binding cassette (ABC) superfamily, major facilitator superfamily (MFS), resistance-nodulation-cell division (RND) superfamily, small multidrug resistance (SMR) family, and multidrug and toxin extrusion (MATE) family.

**Figure 1:**
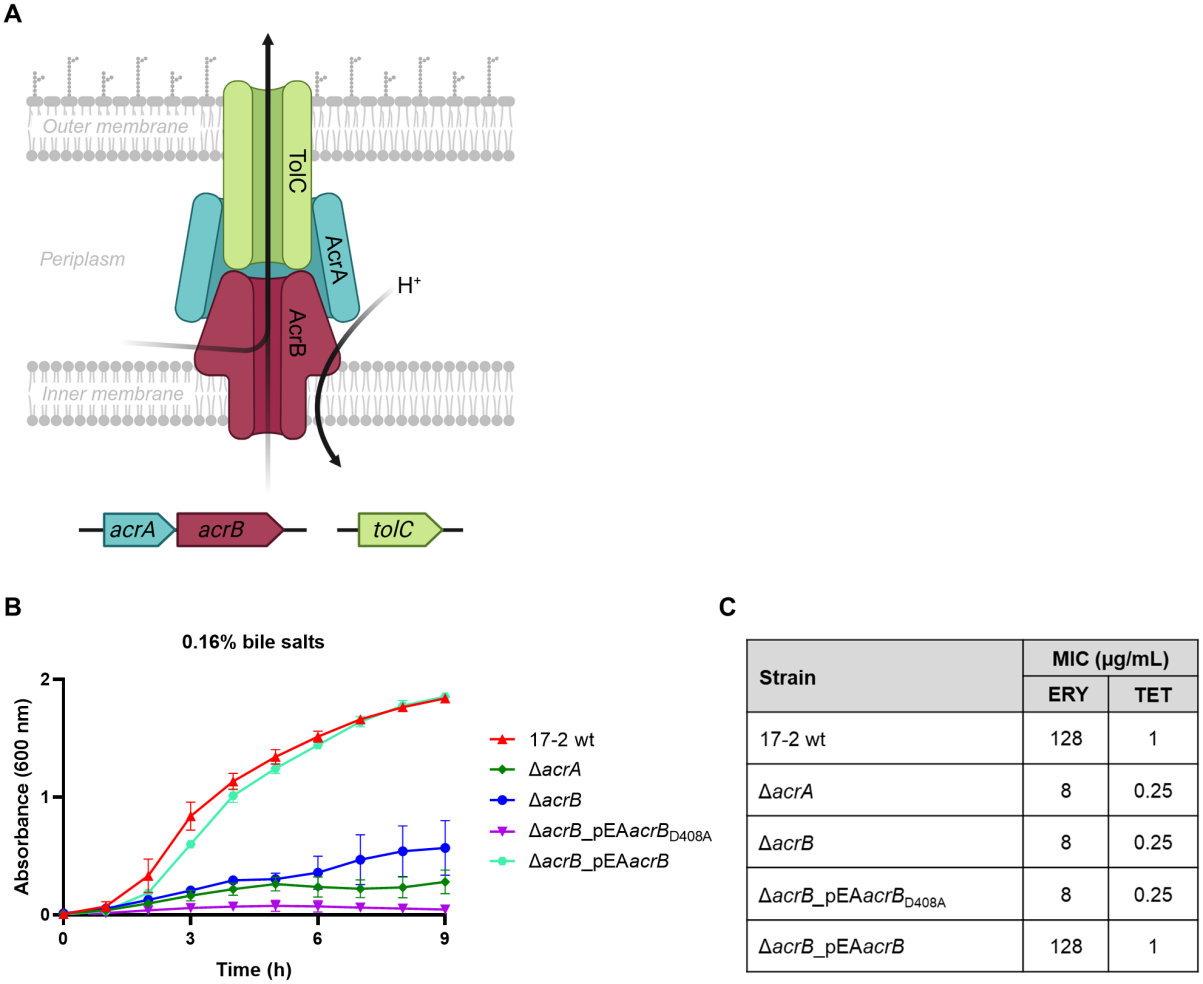
Susceptibility of 17–2 wt and its derivatives to bile salts and antibiotics. **(A)** Schematic representation of the AcrAB-TolC efflux pump and its encoding genes. Created in https://BioRender.com. **(B)** The impact of bile salts was examined on the growth of EAEC 17–2 wt and its derivatives: Δ*acrA*, Δ*acrB*, Δ*acrB_*pEA*acrB*_D408A_ and Δ*acrB_*pEA*acrB*. Bacterial growth was monitored in LB medium with 0.16% (w/v) of a mixture of two bile salts (cholate and deoxycholate) for up to 9 h. The growth curves shown derive from one of three independent experiments, which gave similar results. Error bars represent SD. **(C)** MICs of erythromycin (ERY) and tetracycline (TET) for 17–2 strain and its derivatives. A MIC value that decreases at least 2-fold compared to the wt is considered significant. The results derive from an average of at least three independent experiments.

A set of EAEC 17–2 derivative mutants was generated to study the role of AcrAB in the physiology and infection process of EAEC. These include deletion mutants lacking the periplasmic adaptor protein AcrA or the inner membrane transporter AcrB (**Figure 1A**), as well as complemented strains such as the Δ*acrB*_pEA*acrB*, expressing the wt transporter, or the Δ*acrB*_pEA*acrB*_D408A_, expressing an AcrB protein harbouring the aminoacidic substitution (D408A) that abolishes the efflux activity of the protein (**Supplementary Tables S1**, **S2**) (Wang-Kan et al., 2017). Since AcrB is a very abundant inner membrane protein, the use of this latter 17–2 derivative allowed us to analyse the AcrB efflux function without altering the membrane protein composition (Wang-Kan et al., 2017).

Growth behaviour of EAEC 17–2 wt strain and its derivatives was assessed in different culture conditions. As shown in **Supplementary Figure S1**, the lack of either one of the *acrAB* genes does not affect the growth of 17–2 in LB or DMEM medium. In contrast, when growth was assessed in the presence of bile salts (cholate and deoxycholate) at physiological concentration (0.16% w/v) (**Figure 1B**), measurement of optical density over time showed that both the deletion of *acrA* and *acrB* significantly affected the resistance of 17–2 to bile salts, flattening its growth curve. This phenotype is fully restored in the 17-2 Δ*acrB* mutant transformed with a functional *acrB* gene. Complementation of the Δ*acrB* mutant with the plasmid containing the *acrB*_D408A_ allele produced a phenotype comparable to that of the Δ*acrB* mutant, confirming that the efflux activity and not the simple absence of an abundant inner membrane transporter is responsible for the hypersusceptibility to bile salts. Furthermore, in the 17-2 Δ*acrB*_pEA*acrB*_D408A_ strain, the growth curve appeared even more flattened than in 17–2 strains with Δ*acrA* and Δ*acrB* mutations. Considering the infidelity of the components forming the tripartite efflux pumps (Yamasaki et al., 2011), we can speculate that the AcrA protein produced in 17-2 Δ*acrB*_pEA*acrB*_D408A_ is sequestered by the mutated AcrB transporter to form a non-functional pump, thus preventing it from acting as a periplasmic adaptor for other efflux pumps that may provide partial resistance to bile salts (as in 17-2 Δ*acrA* and 17-2 Δ*acrB*). As expected, considering the role of AcrAB in MDR, we observed that the functionality of this MDR EP is relevant for resistance to erythromycin and tetracycline, two known substrates of AcrAB (**Figure 1C**).

### 3.2 AcrB transporter contributes to biofilm production

Since the ability of EAEC 17–2 to form thick biofilm layers on the intestinal epithelium is a crucial part of its pathogenicity process (Estrada-Garcia et al., 2014), we evaluated the influence of AcrAB on various parameters characterizing bacterial biofilms.

**Figure 2:**
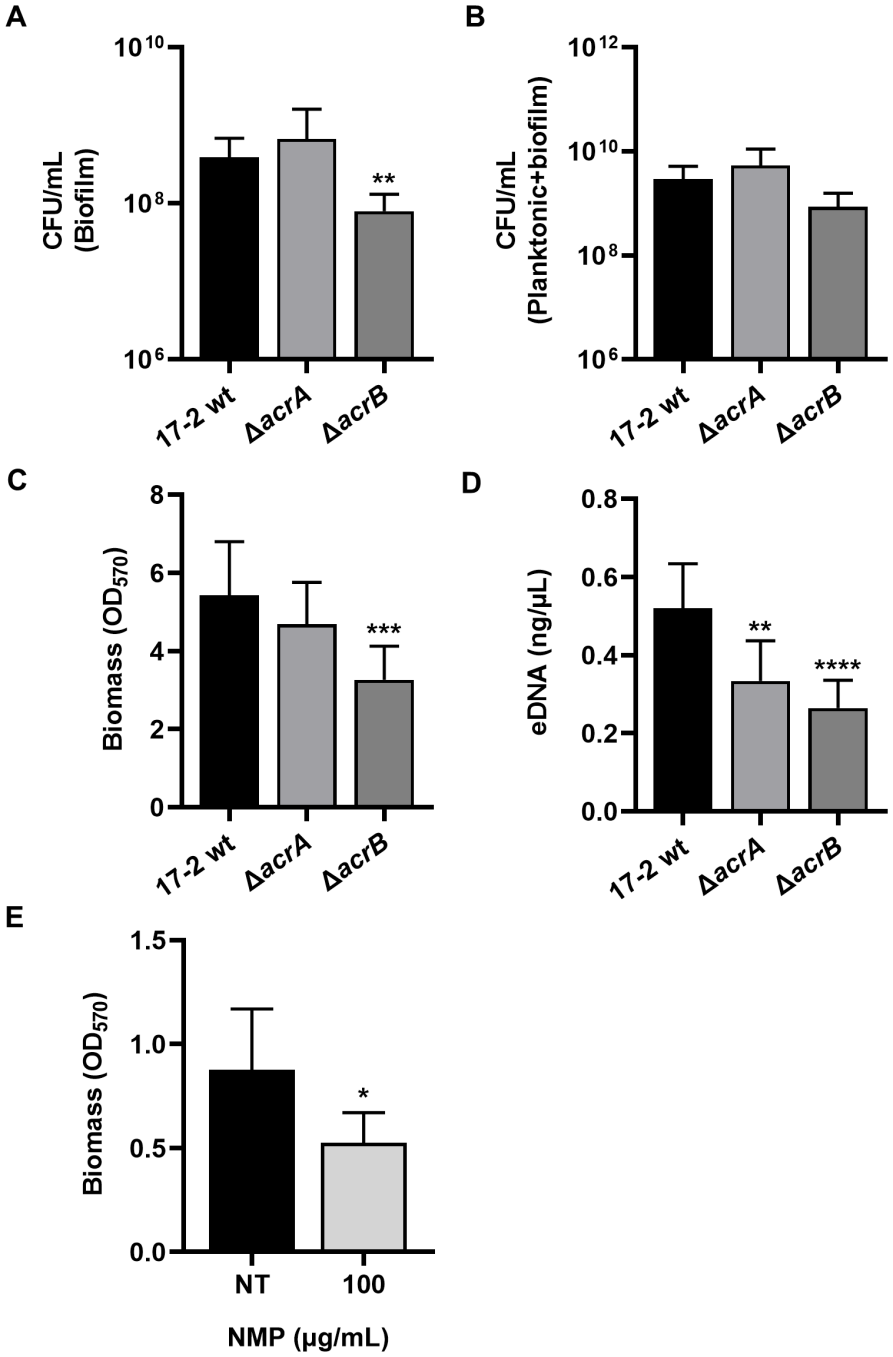
The formation of a mature biofilm in 17–2 is drastically reduced in the absence of AcrB function. The influence of the AcrAB efflux pump on several biofilm parameters was evaluated after 24 h. **(A, B)** The assays were performed with 17–2 wt and its derivatives Δ*acrA* and Δ*acrB*. By enumeration of viable cells, the colony-forming units per millilitre (CFU/mL) of planktonic or biofilm culture were calculated and represented in log_10_ scale. **(C, D)** Biomass and amount of eDNA of the biofilm were studied. **(E)** The effect of NMP on biofilm biomass was assessed by treating 17–2 wt strain with 100 µg/mL of NMP. The non-treated (NT) biofilm of 17–2 wt strain was used as a control. The results shown are the average of three independent experiments. Error bars represent SD. Statistical significance was determined by a one-tailed ANOVA or a two tailed student’s *t*-test and p values are as follows: * *p* < 0.05, ** *p* < 0.01, *** *p* < 0.001, **** *p* < 0.0001.

An initial assessment of biofilm production was performed by counting viable cells (**Figures 2A, B**). The loss of the AcrB transporter caused a significant reduction in the biofilm component compared to the wt strain (**Figure 2A**), despite the total number of bacteria remaining unchanged after 24 h (**Figure 2B**). Consistent with these results, biomass production measured by the crystal violet assay was significantly lower in the Δ*acrB* mutant than in the wt strain (**Figure 2C**). Interestingly, Δ*acrB* biofilms contain less extracellular DNA (eDNA), a key structural component of the biofilm matrix (**Figure 2D**). The composition of the biofilm in terms of protein and amyloid fibres is comparable between wt and defective strains, as well as the metabolic activity, which is not affected at all by the lack of efflux via AcrAB (**Supplementary Figure S2**). Silencing *acrA* had no significant impact on the biofilm parameters analysed, except for eDNA export, reinforcing the hypothesis that this adaptor protein can be easily replaced in the tripartite AcrAB-TolC pump assembly.

To confirm that the role of AcrB in biofilm production is closely linked to the extrusion mediated by this transporter protein, we used 1-(1-naphthyl-methyl)-piperazine (NMP), an AcrB efflux pump inhibitor that acts by mimicking the action of an EP substrate, thus preventing normal substrate extrusion (Bohnert and Kern, 2005). NMP treatment was carried out for 24 hours at 100 µg/mL, a concentration that does not affect the growth of EAEC 17-2 (**Supplementary Figure S3**). Biomass measurement revealed that the inhibition of efflux via AcrB by NMP caused a drastic reduction of the biofilm formation (**Figure 2E**). Consistent with the results obtained with the Δ*acrB* mutant (**Figures 2A-D**), this strongly indicates that the efflux activity associated with AcrB is required to form a thick biofilm layer.

### 3.3 Mutations of the *acrB* gene result in a disorganized aggregative adherence pattern

EAECs are recognized for their characteristic aggregative adherence pattern on HEp-2 cells. This pattern depends on both the self-agglutination of the bacterial cells with each other and their interaction with the epithelial cells (Nataro, 2005; Croxen and Finlay, 2010; Estrada-Garcia et al., 2014). To test whether AcrAB contributed to this phenotype, we performed cell adherence assays on the human epithelial cell line HEp-2. After 24 hours of infection with the 17–2 wt strain, its derivatives Δ*acrA* and Δ*acrB*, and the complemented strains Δ*acrB*_pEA*acrB* and Δ*acrB*_pEA*acrB*_D408A_, samples were fixed and stained with DAPI to label bacterial and cellular DNA, and Evans Blue to stain the cell cytoplasm.

**Figure 3:**
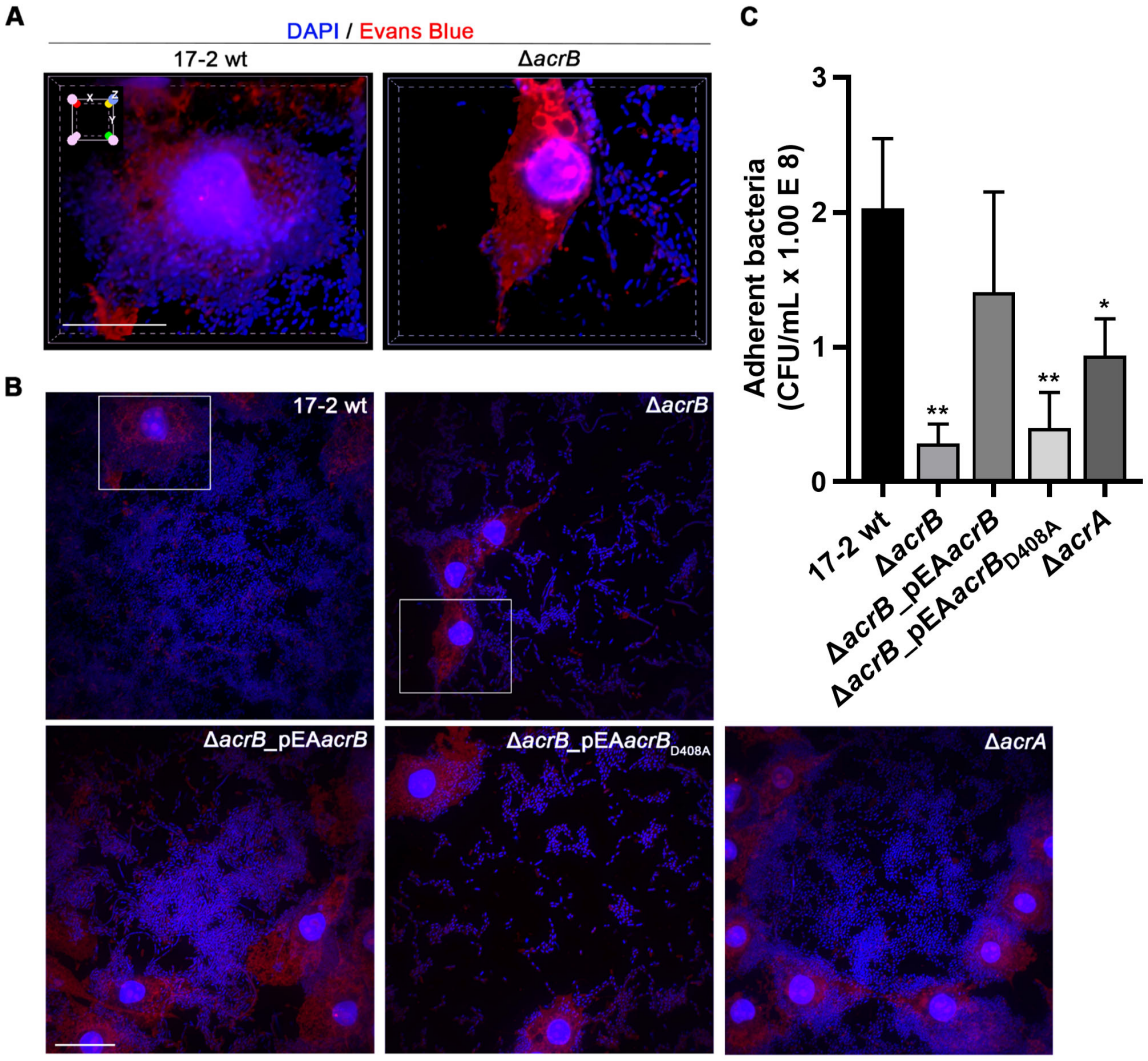
The AcrB transporter is crucial for EAEC aggregative adherence to HEp-2 cells. **(A)** Confocal spinning disk fluorescence microscopy images reconstructed in 3D, showing single HEp-2 cells infected with strains 17–2 and Δ*acrB*. The 3D volume view function of NIS Elements (see Methods) was used to obtain a top-to-bottom view and the x-y-z cube superimposed on top left indicates the three-dimensionality of the sample. Scale bar: 20 µm. **(B)** 2D field images of the aggregative adherence assay of 17–2 wt and its derivatives (Δ*acrB*, Δ*acrB_*pEA*acrB*, Δ*acrB_*pEA*acrB*_D408A_ and Δ*acrA*) to HEp-2 cells. Scale bar: 20 µm. Framed cells correspond to those in panel (A) For both panel A and B samples were fixed 24 h post-infection and stained with 4’,6-diamino-2-phenylindole (DAPI, blue) to visualise DNA of both cells and bacteria, and with Evans Blue (red) to visualise cellular cytoplasm. Representative images of at least three experiments are shown. **(C)** Quantitative adherence assays. HEp-2 cells were infected with 17–2 wt and its derivatives and after 24 h, adherent bacteria were detached by Triton 1% treatment and plated to count viable bacteria (CFU/mL). The results derive from an average of at least three independent experiments. Error bars represent SD. Statistical significance was determined by a one-way ANOVA and p values are as follows: * *p* < 0.05, ** *p* < 0.01.

Confocal microscopy images presented in **Figure 3A** show 3D reconstructions of HEp-2 cells infected with strains 17–2 wt and Δ*acrB*. The wt strain exhibits the “stacked brick” adhesion pattern, with the bacterial cells massively adhering to each other as well as to the cell surface (left panel). Interestingly, the mutant lacking AcrB exhibits a completely different pattern (right panel). Indeed, compared to the wt strain, it appears incapable of forming large aggregates with each other and on the epithelial cell, with an increased dispersion in small clusters over the entire surface of the slide, clearly appreciable in the corresponding 2D panel shown in **Figure 3B**. The aggregative pattern is correctly restored in the complemented strain with a functional AcrB transporter (**Figure 3B**, lower left panel). Conversely, the expression of the AcrB D408A mutated form was unable to functionally complement the defective adherence phenotype shown by Δ*acrB* (**Figure 3B**, lower mid panel). In agreement with previous results, the strain lacking AcrA exhibits an adhesion phenotype similar to that of wt (**Figure 3B**, lower right panel), indicating that this MDR EP may also function in the absence of this component, perhaps utilising other periplasmic adaptors.

Following 24 hours of infection, adherent bacteria were also quantified by viable counts (**Figure 3C**). The results demonstrated that the Δ*acrB* and Δ*acrB*_pEA*acrB*_D408A_ strains are drastically incapable of adherence, suggesting that a functional AcrB transporter is essential for the aggregative adhesion of EAEC.

### 3.4 Efflux via the AcrAB pump affects the *in vivo* virulence of EAEC 17-2

**Figure 4:**
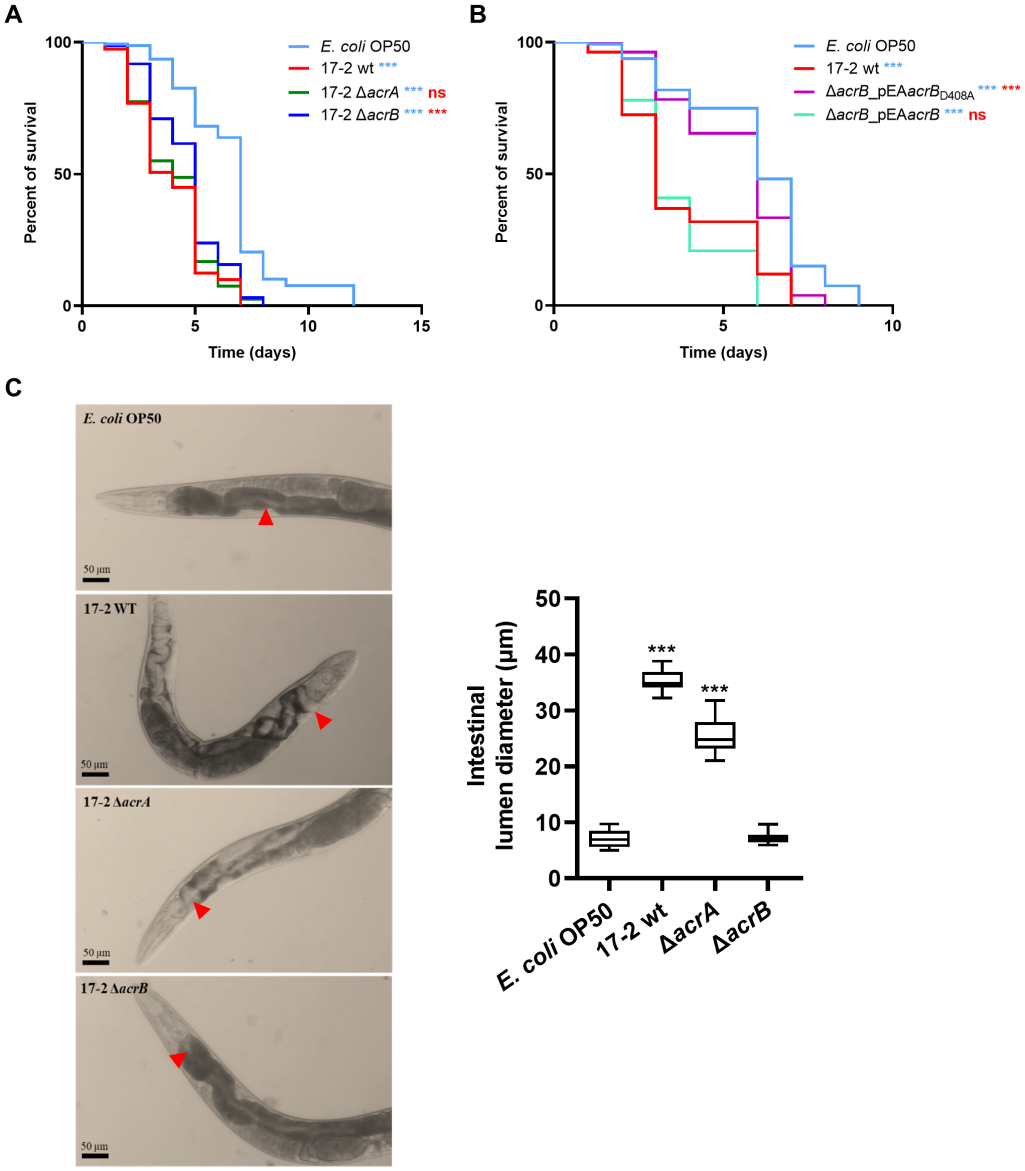
Lack of AcrB makes EAEC less virulent in *C. elegans*. **(A, B)** Kaplan-Meier survival plots were used to compare the survival of worms fed on *E. coli* OP50 (control, cyan) and EAEC strains 17–2 wt (red), 17–2 *acrA* (green), 17-2 Δ*acrB* (blue), Δ*acrB* Δ*acrB_*pEA*acrB*_D408A_ (purple) and Δ*acrB_*pEA*acrB* (bright green). Each experiment involved 80 worms for each strain. Results are representative of at least three independent experiments. Statistical differences between groups were assessed using the log-rank (Mantel-Cox) test. Cyan symbols refer to comparisons with *E. coli* OP50, red symbols refer to comparisons with 17–2 wt (*** *p* < 0.001; ns = non-significant). **(C)** Representative images of individual fixed nematodes were acquired in bright field 3 days after the start of the diet with *E. coli* 0P50 (control) or 17–2 and its derivatives. The diameter of the lumen region immediately after the pharynx is indicated with red arrows and measured on 10 different worms as shown in the graph on the right. Results are the average of at least three independent experiments. Error bars represent SD. Statistical significance was determined by a one-way ANOVA (*** *p* < 0.001).

Using the nematode *C. elegans* as a model to quantitatively evaluate differences in EAEC virulence (Hwang et al., 2010), we assessed the role of the AcrAB efflux pump in the virulence of EAEC 17–2 *in vivo*. Specifically, we fed a synchronous population of worms with either the wild-type strain 17–2 or its derivatives, compared to nematodes fed with a commensal *E. coli* strain (OP50), the standard diet. The number of dead worms was then recorded daily. In line with previous observations (Hwang et al., 2010; Bojer et al., 2012), we found that 17–2 wt is significantly more lethal to worms than OP50 (**Figure 4A**). However, the loss of AcrB transporter caused a significant reduction in pathogenicity for the nematode (*p* = 0.001), with a median lethal time (LT50) of 5 days for 17-2 Δ*acrB* compared to 3 days for 17–2 wt. In contrast, the 17-2 Δ*acrA* mutant appeared just as lethal as the wt strain. Notably, the 17–2 strain harbouring an efflux-defective AcrB protein (AcrB D408A) is even less virulent than the Δ*acrB* strain, with an LT50 similar to that of animals fed with OP50, likely due to its ability to sequester AcrA, making it unavailable for other transporters (**Figure 4B**).

The relevance of AcrB in the infection efficiency of *C. elegans* is evident from the evaluation of the worm gut colonization after three days of feeding with 17–2 wt, Δ*acrA* or Δ*acrB* strains (**Figure 4C**), by measuring the enlargement of the intestinal lumen, a classic marker of nematode stress due to infection by pathogenic bacteria. Indeed, the right panel of **Figure 4C** shows that the lumen diameter ranges between 25 and 35 µm when the worm is fed with the wt strain or its Δ*acrA* derivative. On the contrary, the lumen of the nematode fed with the Δ*acrB* strain appears quite comparable to that colonised by the non-pathogenic *E. coli* OP50, with a diameter of less than 10 µm. Altogether, these data confirm the previous observations on the role of AcrAB and highlight the relevance of the AcrB transporter in the pathogenicity *in vivo*.

## 4 Discussion

The importance of the multidrug-resistant AcrAB efflux pump in the lifestyle of gut pathogens is becoming increasingly evident, as it plays a crucial role in antibiotic resistance, stress adaptation, and host colonization (Buckley et al., 2006; Wang-Kan et al., 2017; Coluccia et al., 2023; Fanelli et al., 2023). In this study, we revealed a novel facet of AcrAB’s role influencing the virulence of the enteroaggregative *Escherichia coli* strain 17-2. Indeed, the data we present demonstrate that AcrAB contributes to the bacterial aggregative phenotype and impacts biofilm formation, both key features of EAEC pathogenesis, thereby shaping the EAEC 17–2 virulence in the *C. elegans in vivo* model. Moreover, here we highlight a specific requirement for the AcrB transporter in the full expression of EAEC 17–2 pathogenicity.

The pathogenesis of EAEC is characterized by the adhesion and aggregation of EAEC to intestinal epithelium with the formation of a dense biofilm followed by mucosal damage, the latter exacerbated by the secretion of one or more cytotoxins (Flores and Okhuysen, 2009). Among the several roles experimentally attributed to AcrAB-TolC EP there is that played in biofilm formation (Alav et al., 2018). Actually, by using the EAEC 17–2 strain deleted of *acrA* or *acrB* component we observed an alteration of the various biofilm parameters evaluated, compared to the wt strain, although with different contributions of the two EP components. In particular, we found that the lack of either AcrA or AcrB significantly affects the accumulation of eDNA in the 17–2 biofilm matrix, a polyvalent molecule in the biofilms of many bacterial species, used for communication, nutrition, genetic exchange and as a structural element that promotes cell-cell adhesion and biofilm stability (Ibáñez de Aldecoa et al., 2017; Campoccia et al., 2021). It is therefore possible to speculate that the observed loss of aggregation in the Δ*acrB* mutant may reflect a failure to accumulate sufficient eDNA to sustain the typical “stacked-brick” architecture of EAEC biofilms. Although this study did not investigate the specific substrate exported by AcrAB in EAEC, the results regarding the reduction of eDNA in the strain defective for AcrA or AcrB components raises the possibility that eDNA, or molecules involved in its release or stabilization, could be exported through the AcrAB efflux pump. Alternatively, AcrAB may influence eDNA accumulation indirectly, by modulating membrane stress responses, cell envelope composition, or the production of outer membrane vesicles (OMVs), which are known vehicles for eDNA in other Gram-negative species (Allesen-Holm et al., 2006; Potapova et al., 2024). Notably, only the loss of the AcrB transporter leads to a significant reduction of biofilm biomass. We were able to replicate this result simply by interfering with the efflux activity of AcrB through the treatment of the wild-type EAEC 17–2 strain with the specific inhibitor NMP (Bohnert and Kern, 2005), suggesting a more prominent role of the inner membrane transporter in biofilm formation.

The AcrB implication in biofilm formation prompted us to study this interesting phenotype in human epithelial cell infection experiments. The fluorescence images obtained following the infection of HEp-2 cells further highlighted the cruciality of AcrB efflux activity in EAEC to ensure the aggregative adherence pattern. Indeed, we found that, even after 24 hours of infection, the mutant defective in the AcrB component had a reduced ability to aggregate, both with each other and with epithelial cells. Additionally, in line with the results obtained with the NMP inhibitor, the mutant expressing the unfunctional AcrB showed a pattern of aggregative adhesion comparable to that of the deletion mutant, further underlining the relevance of the AcrB transporter in the early stage of EAEC pathogenesis.

The impact of AcrB in EAEC virulence was further demonstrated *in vivo* by using the nematode *C. elegans* as a model, which allowed us to mimic the physiological condition of the gastrointestinal tract infection. The *C. elegans* worm population was colonised and killed within five days when fed with 17–2 or with the strain deleted of the periplasmic adaptor protein AcrA (17-2 Δ*acrA*). Conversely, when fed with the strain lacking the AcrB transporter (17-2 Δ*acrB*), their life cycle was extended by one day, and more than 50% of the worms survived until day five. Remarkably, feeding the worm with an AcrB D408A loss-of-efflux-function mutant further prolonged the *C. elegans* life, leading to hypothesize a dominant negative function of the AcrB mutated version, at least in this specific assay, and further strengthening the pivotal role of the AcrB transporter.

AcrAB belongs to the RND superfamily and consists of an inner membrane transporter, AcrB, and a periplasmic adaptor protein (PAP), AcrA. To expel substrates into the extracellular environment, it also requires an outer membrane channel. Like many other tripartite EPs, including members of the RND family, AcrAB utilizes TolC to perform its efflux function. Previous data already suggested an involvement of the efflux network in the EAEC pathogenicity. In fact, Imuta and co-workers demonstrated that TolC plays an important role in the aggregation and adhesion phenotype of the EAEC 042 strain, as well as in biofilm formation, possibly releasing diffusible compounds (Imuta et al., 2008). Tempting to identify a specific EP, the authors also investigated the role of AcrA, finding it dispensable for the aggregation of EAEC. We obtained very similar results, as we proved that AcrA is unnecessary also for biofilm formation and, more importantly, for EAEC pathogenicity *in vivo.* Although the periplasmic protein AcrA is shared by other EPs, i.e. AcrAD, it can be substituted in tripartite pump assembly, as in the case of AcrAB-TolC (Yamasaki et al., 2011), thus explaining the phenotype of the *acrA* deletion mutant described (Imuta et al., 2008; present work). However, the Δ*acrA* mutant shows an increased sensitivity to erythromycin, tetracycline and bile salts. This can be explained considering that the accumulation of toxic substrates in the Δ*acrA* mutant may impact the susceptibility to antibiotic stress and bile salts, but not interfere severely with virulence processes. The data reported here support a specific role for the AcrB transporter, whose activity is as essential for EAEC virulence as that of TolC, making it, reasonably, the specific partner of TolC in determining the full pathogenicity of EAEC.

Enteroaggregative *E. coli* is increasingly recognized as a leading cause of acute diarrhea in children, as well as a major agent of traveler’s diarrhea worldwide. Moreover, it is also capable of provoking extraintestinal infections, including systemic infections (Estrada-Garcia and Navarro-Garcia, 2012; Hebbelstrup Jensen et al., 2014). This, together with the growing prevalence of antimicrobial resistance, particularly multidrug resistance, among EAEC isolates, poses an increasing public health concern (Joffré and Iñiguez Rojas, 2020). Therefore, it becomes increasingly necessary to develop alternative strategies for the treatment of these pathogens. Drugs that interfere with bacterial virulence represent a viable alternative to antibiotic therapy (Dickey et al., 2017). Our results support the hypothesis that MDR EP components, in particular AcrB, may represent valuable targets for this type of strategy. Inhibiting AcrB affects not only antibiotic resistance but also key virulence traits such as aggregation and biofilm formation. By linking efflux pump activity to key pathogenic mechanisms, our study highlights the potential of AcrAB as a dual-function therapeutic target and opens the way to new strategies for managing drug resistant EAEC infections.

## Data Availability

The raw data supporting the conclusions of this article will be made available by the authors, without undue reservation.

## References

[B1] Alav I. Kobylka J. Kuth M. S. Pos K. M. Picard M. Blair J. M. A.. (2021). Structure, assembly, and function of tripartite efflux and type 1 secretion systems in gram-negative bacteria. Chem. Rev. 121, 5479–5596. doi: 10.1021/acs.chemrev.1c00055, PMID: 33909410 PMC8277102

[B2] Alav I. Sutton J. M. Rahman K. M. (2018). Role of bacterial efflux pumps in biofilm formation. J. Antimicrobial Chemotherapy 73, 2003–2020. doi: 10.1093/jac/dky042, PMID: 29506149

[B3] Alcalde-Rico M. Hernando-Amado S. Blanco P. Martínez J. L. (2016). Multidrug efflux pumps at the crossroad between antibiotic resistance and bacterial virulence. Front. Microbiol. 7. doi: 10.3389/fmicb.2016.01483, PMID: 27708632 PMC5030252

[B4] Allesen-Holm M. Barken K. B. Yang L. Klausen M. Webb J. S. Kjelleberg S.. (2006). A characterization of DNA release in *Pseudomonas aeruginosa* cultures and biofilms. Mol. Microbiol. 59, 1114–1128. doi: 10.1111/j.1365-2958.2005.05008.x, PMID: 16430688

[B5] Berry A. A. Yang Y. Pakharukova N. Garnett J. A. Lee W. Cota E.. (2014). Structural Insight into Host Recognition by Aggregative Adherence Fimbriae of Enteroaggregative Escherichia coli. PloS Pathog. 10, e1004404. doi: 10.1371/journal.ppat.1004404, PMID: 25232738 PMC4169507

[B6] Blair J. M. A. Webber M. A. Baylay A. J. Ogbolu D. O. Piddock L. J. V. (2015). Molecular mechanisms of antibiotic resistance. Nat. Rev. Microbiol. 13, 42–51. doi: 10.1038/nrmicro3380, PMID: 25435309

[B7] Blanco P. Hernando-Amado S. Reales-Calderon J. Corona F. Lira F. Alcalde-Rico M.. (2016). Bacterial multidrug efflux pumps: much more than antibiotic resistance determinants. Microorganisms 4, 14. doi: 10.3390/microorganisms4010014, PMID: 27681908 PMC5029519

[B8] Bohnert J. A. Kern W. V. (2005). Selected arylpiperazines are capable of reversing multidrug resistance in *escherichia coli* overexpressing RND efflux pumps. Antimicrob. Agents Chemother. 49, 849–852. doi: 10.1128/AAC.49.2.849-852.2005, PMID: 15673787 PMC547223

[B9] Bojer M. S. Jakobsen H. Struve C. Krogfelt K. A. Løbner-Olesen A. (2012). Lack of the RNA chaperone Hfq attenuates pathogenicity of several Escherichia coli pathotypes towards Caenorhabditis elegans. Microbes Infect. 14, 1034–1039. doi: 10.1016/j.micinf.2012.06.002, PMID: 22713744

[B10] Buckley A. M. Webber M. A. Cooles S. Randall L. P. La Ragione R. M. Woodward M. J.. (2006). The AcrAB-TolC efflux system of Salmonella enterica serovar Typhimurium plays a role in pathogenesis. Cell Microbiol. 8, 847–856. doi: 10.1111/j.1462-5822.2005.00671.x, PMID: 16611233

[B11] Campoccia D. Montanaro L. Arciola C. R. (2021). Extracellular DNA (eDNA). A major ubiquitous element of the bacterial biofilm architecture. Int. J. Mol. Sci. 22, 9100. doi: 10.3390/ijms22169100, PMID: 34445806 PMC8396552

[B12] Choong F. X. Bäck M. Fahlén S. Johansson L. B. Melican K. Rhen M.. (2016). Real-time optotracing of curli and cellulose in live Salmonella biofilms using luminescent oligothiophenes. NPJ Biofilms Microbiomes 2, 16024. doi: 10.1038/npjbiofilms.2016.24, PMID: 28721253 PMC5515270

[B13] Coluccia M. Béranger A. Trirocco R. Fanelli G. Zanzi F. Colonna B.. (2023). Role of the MDR efflux pump acrAB in epithelial cell invasion by shigella flexneri. Biomolecules 13, 823. doi: 10.3390/biom13050823, PMID: 37238693 PMC10216353

[B14] Cravioto A. Gross R. J. Scotland S. M. Rowe B. (1979). An adhesive factor found in strains ofEscherichia coli belonging to the traditional infantile enteropathogenic serotypes. Curr. Microbiol. 3, 95–99. doi: 10.1007/BF02602439

[B15] Croxen M. A. Finlay B. B. (2010). Molecular mechanisms of Escherichia coli pathogenicity. Nat. Rev. Microbiol. 8, 26–38. doi: 10.1038/nrmicro2265, PMID: 19966814

[B16] Datsenko K. A. Wanner B. L. (2000). One-step inactivation of chromosomal genes in *Escherichia coli* K-12 using PCR products. Proc. Natl. Acad. Sci. 97, 6640–6645. doi: 10.1073/pnas.120163297, PMID: 10829079 PMC18686

[B17] Dickey S. W. Cheung G. Y. C. Otto M. (2017). Different drugs for bad bugs: antivirulence strategies in the age of antibiotic resistance. Nat. Rev. Drug Discov. 16, 457–471. doi: 10.1038/nrd.2017.23, PMID: 28337021 PMC11849574

[B18] Du D. Wang Z. James N. R. Voss J. E. Klimont E. Ohene-Agyei T.. (2014). Structure of the AcrAB–TolC multidrug efflux pump. Nature 509, 512–515. doi: 10.1038/nature13205, PMID: 24747401 PMC4361902

[B19] Du D. Wang-Kan X. Neuberger A. van Veen H. W. Pos K. M. Piddock L. J. V.. (2018). Multidrug efflux pumps: structure, function and regulation. Nat. Rev. Microbiol. 16, 523–539. doi: 10.1038/s41579-018-0048-6, PMID: 30002505

[B20] Ellis S. J. Crossman L. C. McGrath C. J. Chattaway M. A. Hölken J. M. Brett B.. (2020). Identification and characterisation of enteroaggregative Escherichia coli subtypes associated with human disease. Sci. Rep. 10, 7475. doi: 10.1038/s41598-020-64424-3, PMID: 32366874 PMC7198487

[B21] Estrada-Garcia T. Navarro-Garcia F. (2012). Enteroaggregative *Escherichia coli* pathotype: a genetically heterogeneous emerging foodborne enteropathogen. FEMS Immunol. Med. Microbiol. 66, 281–298. doi: 10.1111/j.1574-695X.2012.01008.x, PMID: 22775224

[B22] Estrada-Garcia T. Perez-Martinez I. Bernal-Reynaga R. Zaidi M. B. (2014). Enteroaggregative escherichia coli: A pathogen bridging the north and south. Curr. Trop. Med. Rep. 1, 88–96. doi: 10.1007/s40475-014-0018-7, PMID: 24892007 PMC4039296

[B23] Fanelli G. Pasqua M. Prosseda G. Grossi M. Colonna B. (2023). AcrAB efflux pump impacts on the survival of adherent-invasive Escherichia coli strain LF82 inside macrophages. Sci. Rep. 13, 2692. doi: 10.1038/s41598-023-29817-0, PMID: 36792672 PMC9931695

[B24] Feng Y. Chen Z. Liu S.-L. (2011). Gene decay in shigella as an incipient stage of host-adaptation. PloS One 6, e27754. doi: 10.1371/journal.pone.0027754, PMID: 22110755 PMC3218036

[B25] Flores J. Okhuysen P. C. (2009). Enteroaggregative Escherichia coli infection. Curr. Opin. Gastroenterol. 25, 8–11. doi: 10.1097/MOG.0b013e32831dac5e, PMID: 19114769

[B26] Hebbelstrup Jensen B. Olsen K. E. P. Struve C. Krogfelt K. A. Petersen A. M. (2014). Epidemiology and clinical manifestations of enteroaggregative escherichia coli. Clin. Microbiol. Rev. 27, 614–630. doi: 10.1128/CMR.00112-13, PMID: 24982324 PMC4135892

[B27] Henderson P. J. F. Maher C. Elbourne L. D. H. Eijkelkamp B. A. Paulsen I. T. Hassan K. A. (2021). Physiological functions of bacterial “Multidrug” Efflux pumps. Chem. Rev. 121, 5417–5478. doi: 10.1021/acs.chemrev.0c01226, PMID: 33761243

[B28] Hwang J. Mattei L. M. VanArendonk L. G. Meneely P. M. Okeke I. N. (2010). A Pathoadaptive Deletion in an Enteroaggregative *Escherichia coli* Outbreak Strain Enhances Virulence in a *Caenorhabditis elegans* Model. Infect. Immun. 78, 4068–4076. doi: 10.1128/IAI.00014-10, PMID: 20584976 PMC2937471

[B29] Ibáñez de Aldecoa A. L. Zafra O. González-Pastor J. E. (2017). Mechanisms and regulation of extracellular DNA release and its biological roles in microbial communities. Front. Microbiol. 8. doi: 10.3389/fmicb.2017.01390, PMID: 28798731 PMC5527159

[B30] Imuta N. Nishi J. Tokuda K. Fujiyama R. Manago K. Iwashita M.. (2008). The *Escherichia coli* Efflux Pump TolC Promotes Aggregation of Enteroaggregative E. coli 042. Infect. Immun. 76, 1247–1256. doi: 10.1128/IAI.00758-07, PMID: 18160483 PMC2258836

[B31] Joffré E. Iñiguez Rojas V. (2020). Molecular epidemiology of enteroaggregative escherichia coli (EAEC) isolates of hospitalized children from Bolivia reveal high heterogeneity and multidrug-resistance. Int. J. Mol. Sci. 21, 9543. doi: 10.3390/ijms21249543, PMID: 33334000 PMC7765457

[B32] Li X.-Z. Plésiat P. Nikaido H. (2015). The challenge of efflux-mediated antibiotic resistance in gram-negative bacteria. Clin. Microbiol. Rev. 28, 337–418. doi: 10.1128/CMR.00117-14, PMID: 25788514 PMC4402952

[B33] Murakami S. Nakashima R. Yamashita E. Yamaguchi A. (2002). Crystal structure of bacterial multidrug efflux transporter AcrB. Nature 419, 587–593. doi: 10.1038/nature01050, PMID: 12374972

[B34] Nataro J. P. (2005). Enteroaggregative Escherichia coli pathogenesis. Curr. Opin. Gastroenterol. 21, 4–8. 15687877

[B35] Nikaido H. Pagès J.-M. (2012). Broad-specificity efflux pumps and their role in multidrug resistance of Gram-negative bacteria. FEMS Microbiol. Rev. 36, 340–363. doi: 10.1111/j.1574-6976.2011.00290.x, PMID: 21707670 PMC3546547

[B36] Nishino K. Yamaguchi A. (2001). Analysis of a complete library of putative drug transporter genes in *escherichia coli* . J. Bacteriol 183, 5803–5812. doi: 10.1128/JB.183.20.5803-5812.2001, PMID: 11566977 PMC99656

[B37] Padilla E. Llobet E. Doménech-Sánchez A. Martínez-Martínez L. Bengoechea J. A. Albertí S. (2010). *Klebsiella pneumoniae* acrAB efflux pump contributes to antimicrobial resistance and virulence. Antimicrob. Agents Chemother. 54, 177–183. doi: 10.1128/AAC.00715-09, PMID: 19858254 PMC2798511

[B38] Pasqua M. Bonaccorsi di Patti M. C. Fanelli G. Utsumi R. Eguchi Y. Trirocco R.. (2021). Host - bacterial pathogen communication: the wily role of the multidrug efflux pumps of the MFS family. Front. Mol. Biosci. 8. doi: 10.3389/fmolb.2021.723274, PMID: 34381818 PMC8350985

[B39] Pérez A. Poza M. Fernández A. del Carmen Fernández M. Mallo S. Merino M.. (2012). Involvement of the acrAB-tolC efflux pump in the resistance, fitness, and virulence of enterobacter cloacae. Antimicrob. Agents Chemother. 56, 2084–2090. doi: 10.1128/AAC.05509-11, PMID: 22290971 PMC3318359

[B40] Petro C. D. Duncan J. K. Seldina Y. I. Allué-Guardia A. Eppinger M. Riddle M. S.. (2020). Genetic and virulence profiles of enteroaggregative escherichia coli (EAEC) isolated from deployed military personnel (DMP) with travelers’ Diarrhea. Front. Cell Infect. Microbiol. 10. doi: 10.3389/fcimb.2020.00200, PMID: 32509590 PMC7251025

[B41] Piddock L. J. V. (2006). Multidrug-resistance efflux pumps? not just for resistance. Nat. Rev. Microbiol. 4, 629–636. doi: 10.1038/nrmicro1464, PMID: 16845433

[B42] Pokharel P. Dhakal S. Dozois C. M. (2023). The Diversity of Escherichia coli Pathotypes and Vaccination Strategies against This Versatile Bacterial Pathogen. Microorganisms 11, 344. doi: 10.3390/microorganisms11020344, PMID: 36838308 PMC9965155

[B43] Potapova A. Garvey W. Dahl P. Guo S. Chang Y. Schwechheimer C.. (2024). Outer membrane vesicles and the outer membrane protein OmpU govern *Vibrio cholerae* biofilm matrix assembly. mBio 15, e0330423. doi: 10.1128/mbio.03304-23, PMID: 38206049 PMC10865864

[B44] Ravaioli S. Campoccia D. Speziale P. Pietrocola G. Zatorska B. Maso A.. (2020). Various biofilm matrices of the emerging pathogen *Staphylococcus lugdunensis*: exopolysaccharides, proteins, eDNA and their correlation with biofilm mass. Biofouling 36, 86–100. doi: 10.1080/08927014.2020.1716217, PMID: 31985269

[B45] Schifano E. Ficociello G. Vespa S. Ghosh S. Cipollo J. F. Talora C.. (2019). *Pmr-1* gene affects susceptibility of *Caenorhabditis elegans* to *Staphylococcus aureus* infection through glycosylation and stress response pathways’ alterations. Virulence 10, 1013–1025. doi: 10.1080/21505594.2019.1697118, PMID: 31771413 PMC6930020

[B46] Sivori F. Cavallo I. Kovacs D. Guembe M. Sperduti I. Truglio M.. (2022). Role of Extracellular DNA in Dalbavancin Activity against Methicillin-Resistant Staphylococcus aureus (MRSA) Biofilms in Patients with Skin and Soft Tissue Infections. Microbiol. Spectr. 10, e0035122. doi: 10.1128/spectrum.00351-22, PMID: 35416701 PMC9045124

[B47] Sivori F. Cavallo I. Truglio M. De Maio F. Sanguinetti M. Fabrizio G.. (2024). Staphylococcus aureus colonizing the skin microbiota of adults with severe atopic dermatitis exhibits genomic diversity and convergence in biofilm traits. Biofilm 8, 100222. doi: 10.1016/j.bioflm.2024.100222, PMID: 39381779 PMC11460521

[B48] Spaniol V. Bernhard S. Aebi C. (2015). Moraxella catarrhalis AcrAB-OprM Efflux Pump Contributes to Antimicrobial Resistance and Is Enhanced during Cold Shock Response. Antimicrob. Agents Chemother. 59, 1886–1894. doi: 10.1128/AAC.03727-14, PMID: 25583725 PMC4356836

[B49] Teelucksingh T. Thompson L. K. Zhu S. Kuehfuss N. M. Goetz J. A. Gilbert S. E.. (2022). A genetic platform to investigate the functions of bacterial drug efflux pumps. Nat. Chem. Biol. 18, 1399–1409. doi: 10.1038/s41589-022-01119-y, PMID: 36065018

[B50] Wang-Kan X. Blair J. M. A. Chirullo B. Betts J. La Ragione R. M. Ivens A.. (2017). Lack of acrB efflux function confers loss of virulence on *salmonella enterica* serovar typhimurium. mBio 8, e00968-17. doi: 10.1128/mBio.00968-17, PMID: 28720734 PMC5516257

[B51] Webber M. A. Bailey A. M. Blair J. M. A. Morgan E. Stevens M. P. Hinton J. C. D.. (2009). The global consequence of disruption of the acrAB-tolC efflux pump in *salmonella enterica* includes reduced expression of SPI-1 and other attributes required to infect the host. J. Bacteriol 191, 4276–4285. doi: 10.1128/JB.00363-09, PMID: 19411325 PMC2698494

[B52] World Health Organization (2017). Available online at: https://www.who.int/news/item/27-02-2017-who-publishes-list-of-bacteria-for-which-new-antibiotics-are-urgently-needed.

[B53] Yamasaki S. Nagasawa S. Hayashi-Nishino M. Yamaguchi A. Nishino K. (2011). AcrA dependency of the AcrD efflux pump in Salmonella enterica serovar Typhimurium. J. Antibiot (Tokyo) 64, 433–437. doi: 10.1038/ja.2011.28, PMID: 21505470

[B54] Zwama M. Nishino K. (2021). Ever-adapting RND efflux pumps in gram-negative multidrug-resistant pathogens: A race against time. Antibiotics 10, 774. doi: 10.3390/antibiotics10070774, PMID: 34201908 PMC8300642

