# Supplementary material for: The AcrAB efflux pump contributes to the virulence of Enteroaggregative E. coli by influencing the aggregative behavior
Source: Front Cell Infect Microbiol. 2025 Aug 7;15:1633585. doi: 10.3389/fcimb.2025.1633585 (PMC12367648; doi:10.3389/fcimb.2025.1633585)
Supplement: Supplementary file 1 [file Table1.docx]

Supplementary Material

# Supplementary Figures and Tables

## Supplementary Figures


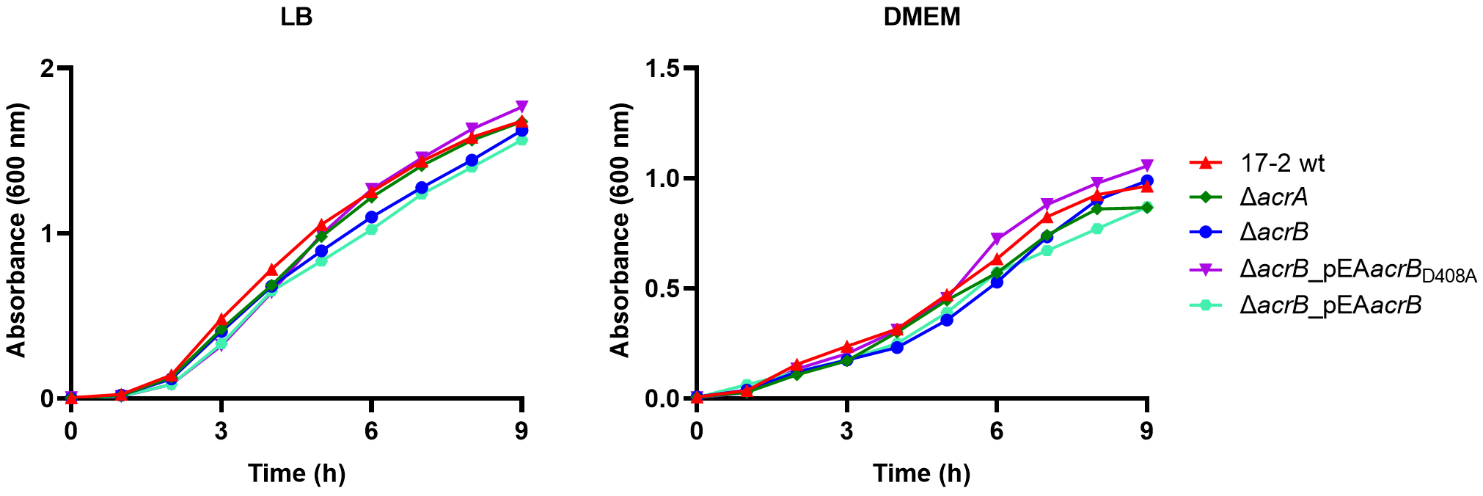


**Supplementary Figure 1.** **Growth curves of 17-2 wt and derivative strains**. EAEC 17-2 and the indicated derivatives were grown in both LB and DMEM medium and the absorbance at 600 nm was measured over time (9 h). The growth curves shown derive from one of three independent experiments, which gave similar results.

**
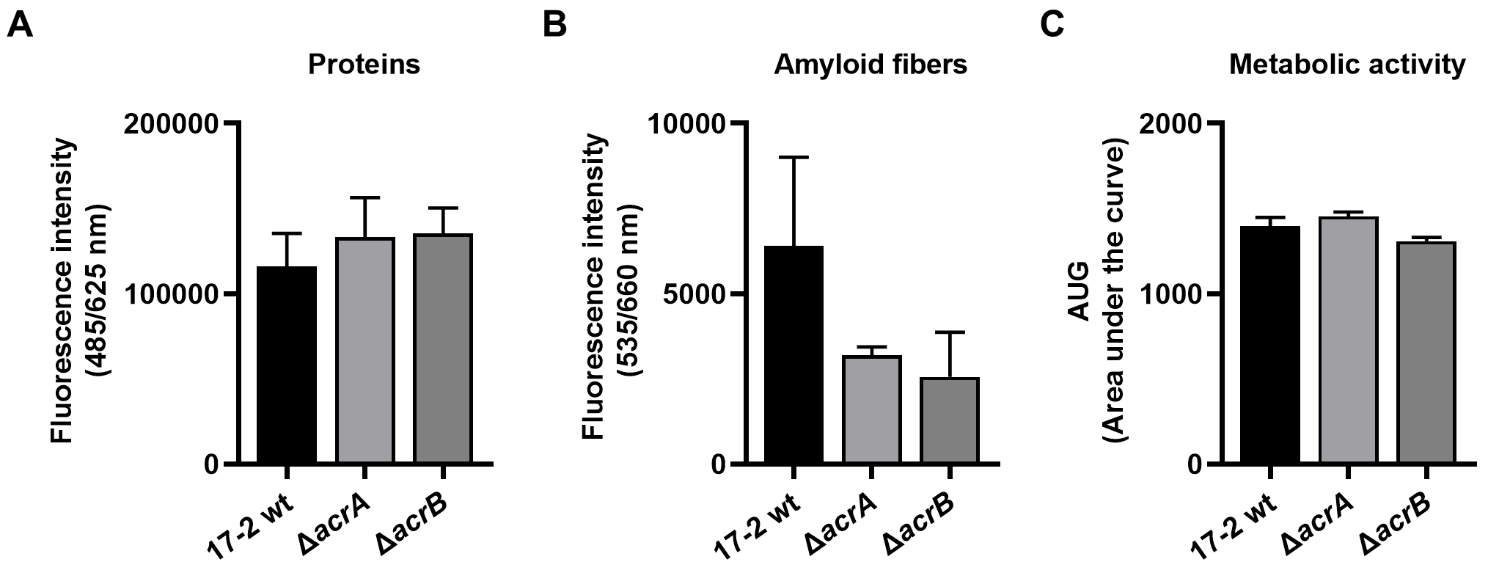
**

**Supplementary Figure 2. Biofilm matrix composition and metabolic activity are unaffected by AcrA or AcrB inactivation.** Quantification of total proteins **(A)**, amyloid fibers **(B)**, and **(C)** the metabolic activity of biofilm-associated cells revealed no significant differences between the 17-2 wt strain and the Δ*acrA* and Δ*acrB* mutants. The results derive from an average of at least three independent experiments. Error bars represent SD.


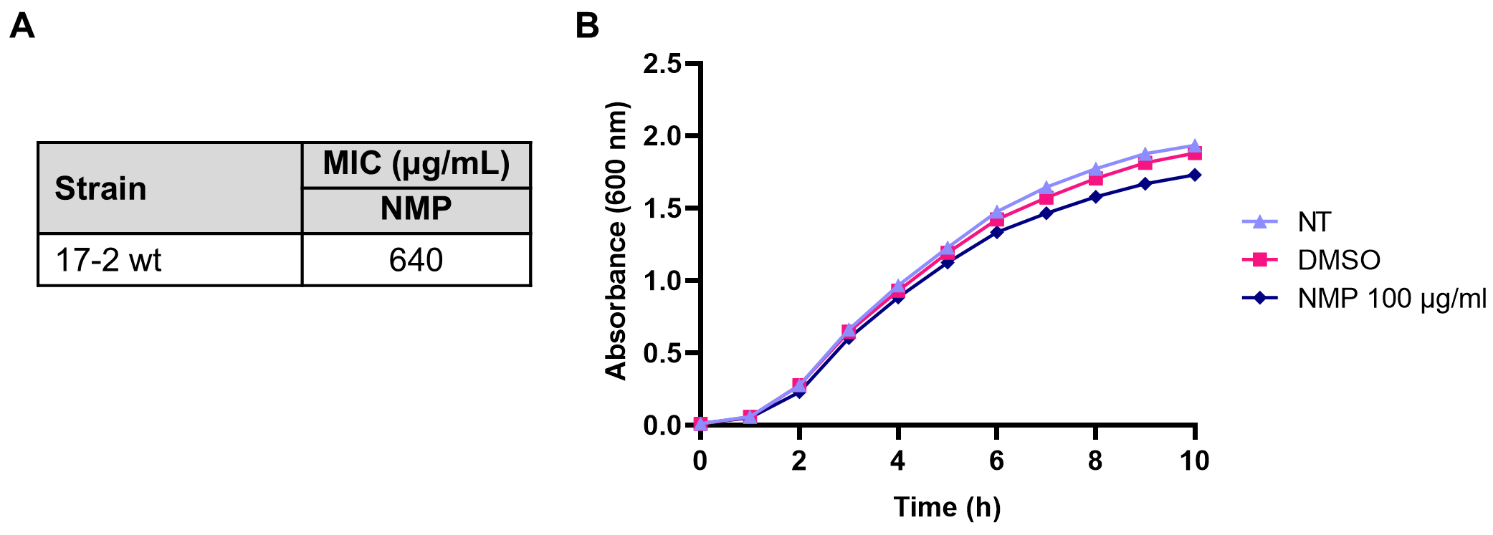


**Supplementary Figure 3. Susceptibility of 17-2 wt strain to NMP. (A)** The MIC of NMP for the 17-2 wt strain was determined and the result shown is based on an average of three independent experiments. In all experiments involving NMP, the concentration used was 100 µg/mL, significantly lower than the MIC value obtained. **(B)** Growth curves of 17-2 wt strain in the presence of 100 µg/mL of 1-(1-naphthylmethyl)-piperazine (NMP) or DMSO (used as NMP solvent). The results shown are representative of one of three independent experiments.

## Supplementary Tables

**Supplementary Table 1. Strains used in this study.**

| **Strains** | **Characteristics** | **Source/Reference** |
| --- | --- | --- |
| 17-2 wt | Strain 17-2 (serotype 03:H2) of EAEC isolated from the diarrheic stool of a child in Santiago, Chile, in 1988 | Nataro et al., 1994 |
| 17-2 *∆acrA* | 17-2 derivative strain defective in *acrA* gene, Km^S^ (Sensitive) | This study |
| 17-2 *∆acrB* | 17-2 derivative strain defective in *acrB* gene, Km^S^ | This study |
| DH10b | F– *mcrA* Δ (*mrr*-*hsdRMS*-*mcrBC*) φ80*lacZ*ΔM15 Δ*lac*X74 *recA*1 *endA*1 *araD*139 Δ(*ara*-*leu*)7697 *galU* *galK* λ– *rpsL*(StrR) *nupG* | Sambrock and Russel, 2001 |
| *E. coli* OP50 | Standard food source for *C. elegans* and negative control in killing assays | Stiernagle, 2006 |

**Supplementary Table 2. Plasmids used in this study.**

| **Plasmid** | **Characteristics** | **Source/References** |
| --- | --- | --- |
| pKD46 | Temperature-sensitive replicon that carried bacteriophage λ red genes (*γ*, *β* and *exo*) under control of arabinose inducible P*araBAD* promoter, Ap^R^ (Resistant) | Datsenko and Wanner, 2000 |
| pKD4 | Template plasmid containing Km resistance gene flanked by FLP recognition target sequences with an adjacent ribosome-binding site, Km^R^, Ap^R^ | Datsenko and Wanner, 2000 |
| pKD13 | Template plasmid carrying a kanamycin resistance gene flanked by FLP recognition target sequences, Km^R^, Ap^R^ | Datsenko and Wanner, 2000 |
| pCP20 | Temperature-sensitive replicon carrying the yeast FLP recombinase gene, Ap^R^ | Datsenko and Wanner, 2000 |
| pGIP7 | pACYC184-derived vector carrying *lacI*-*lac* promoter region, Cm^R^ | Falconi et al., 2001 |
| pEA*acrB* | pGIP7 derivative plasmid carrying the *acrB* gene cloned into BamHI site, Cm^R^ | This study |
| pEA*acrB*_D408A_ | pGIP7 derivative plasmid carrying the *acrB*_D408A_ allele, Cm^R^ | This study |

**Supplementary Table 3. Oligonucleotides used in this study.**

| **Name** | **5’- 3’ sequences** |
| --- | --- |
| **Chromosomal mutations** | |
| AFW | ACTTTTGACCATTGACCAATTTGAAATCGGACACTCGAGGTTTACATATGTGTAGGCTGGAGCTGCTTCG |
| ARV | TAGGCATGTCTTAACGGCTCCTGTTTAAGTTAAGACTTGGACTGTTCAGGCATATGAATATCCTCCTTA |
| BFW | CCTGAACAGTCCAAGTCTTAACTTAAACAGGAGCCGTTAAGACATGCCTATGTAGGCTGGAGCTGCTTCG |
| BRV | TATAAAAAAGGCCGCTTGCGCGGCCTTAGTGATTACACGTTGTATCAATGATTCCGGGGATCCGTCGACC |
| **Construction of plasmids** | |
| **Name** | **5’- 3’ sequences** |
| pEA*acrB*FW | NNNGGATCCATGCCTAATTTCTTTATCGATCGC |
| pEA*acrB*RV | NNNGGATCCCAATGATGATCGACAGTATG |
| pEA*acrB*_D408A_FW | CATCGGCCTGTTGGTGGATGCCGCCATCGTTGTGGTAGAAA |
| pEA*acrB*_D408A_RV | TTTCTACCACAACGATGGCGGCATCCACCAACAGGCCGATGG |

# References

Datsenko, K. A., and Wanner, B. L. (2000). One-step inactivation of chromosomal genes in *Escherichia coli* K-12 using PCR products. *Proceedings of the National Academy of Sciences* 97, 6640–6645. doi: 10.1073/pnas.120163297

Falconi, M., Prosseda, G., Giangrossi, M., Beghetto, E., and Colonna, B. (2001). Involvement of FIS in the H‐NS‐mediated regulation of *virF* gene of *Shigella* and enteroinvasive *Escherichia coli*  ^‡^. *Mol Microbiol* 42, 439–452. doi: 10.1046/j.1365-2958.2001.02646.x

Nataro, J. P., Yikang, D., Yingkang, D., and Walker, K. (1994). AggR, a transcriptional activator of aggregative adherence fimbria I expression in enteroaggregative Escherichia coli. *J Bacteriol* 176, 4691–4699. doi: 10.1128/jb.176.15.4691-4699.1994

Sambrock, J., and Russel, D. W. (2001). *Molecular Cloning: A Laboratory Manual*., 3rd ed. Cold Spring Harbor Laboratory Press. Available at: https://www.researchgate.net/publication/200037138_Molecular_Cloning_A_Laboratory_Manual_3-Volume_Set (Accessed December 30, 2020).

Stiernagle, T. (2006). Maintenance of C. elegans. *WormBook*. doi: 10.1895/wormbook.1.101.1
